# Supplementary material for: Characterization of the TCR β Chain CDR3 Repertoire in Subarachnoid Hemorrhage Patients with Delayed Cerebral Ischemia
Source: Int J Mol Sci. 2020 Apr 29;21(9):3149. doi: 10.3390/ijms21093149 (PMC7246515; doi:10.3390/ijms21093149)
Supplement: Supplementary file 1 [file ijms-21-03149-s001.pdf]

Supplementary data

Figure 1. TCRB CDR3 clonotypes on the day of admisson and DCI are represented as dots and are ranked based on their frequency, from high to low [12].

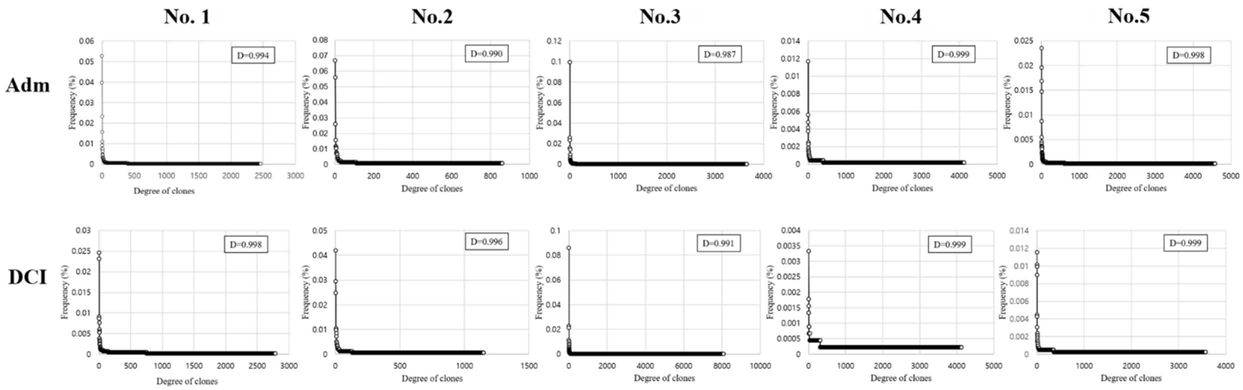

Figure 2. Overlapping sequences of the TCRB CDR3 amino acid in the two different time period of admission and DCI in each patients. The distribution of the top 5 common overlapped amino acid sequences is presented on the right side.

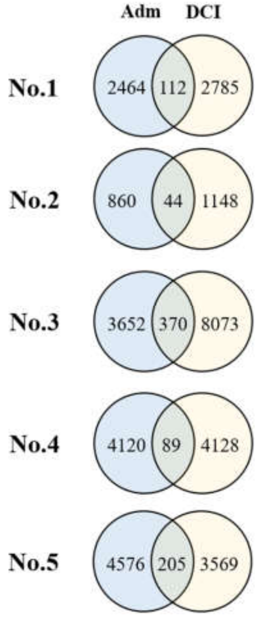

Amino acid	V gene	J gene	Total	Adm*	DCI*
CASSPPGAEGEQYF	TCRBV03-01/03-02	TCRBJ02-07	294	4.29	1.89
CASSRVQGQGQPQHF	TCRBV06-08	TCRBJ01-05	251	3.22	2.01
CASSLAGSGTEAFF	TCRBV07-09	TCRBJ01-01	119	1.9	0.62
CASTAGGGGEQYF	TCRBV05-01	TCRBJ02-07	82	1.29	0.45
CARWLGAVPMNTEAFF	TCRBV02-01	TCRBJ01-01	66	0.61	0.74

AminoAcid	V gene	J gene	Total	Adm*	DCI*
CASRGVGEKLFF	TCRBV28-01	TCRBJ01-04	149	5.27	3.41
CASSESGGITNTGELFF	TCRBV10-02	TCRBJ02-02	71	2.04	2.02
CASSSGTGAYNEQFF	TCRBV05-01	TCRBJ02-01	35	1.24	0.80
CASSQDSYEQYF	TCRBV04-01	TCRBJ02-07	28	0.81	0.80
CASRGLAKGIEQYF	TCRBV27-01	TCRBJ02-07	20	0.56	0.59

AminoAcid	V gene	J gene	Total	Adm*	DCI*
CASSPLTGNTIYF	TCRBV27-01	TCRBJ01-03	1660	7.92	6.89
CASSSSTGYSPLHF	TCRBV27-01	TCRBJ01-06	419	2.06	1.71
CASSTRPGTEAFF	TCRBV13-01	TCRBJ01-01	227	1.15	0.91
CASSGDRGGLYEQYF	TCRBV10-02	TCRBJ02-07	178	0.98	0.68
CASSSSTTSGRAKDTQYF	TCRBV27-01	TCRBJ02-03	128	0.53	0.57

AminoAcid	V gene	J gene	Total	Adm*	DCI*
CASSPGGGPQPQHF	TCRBV09-01	TCRBJ01-05	71	0.97	0.28
CASSSETEGYNEQFF	TCRBV05-06	TCRBJ02-01	26	0.40	0.06
CASSVGDTEAFF	TCRBV09-01	TCRBJ01-01	20	0.21	0.15
CASRSGTSDHEQYF	TCRBV02-01	TCRBJ02-07	19	0.31	0.02
CASSFTGGREKLFF	TCRBV05-05	TCRBJ01-04	15	0.19	0.07

AminoAcid	V gene	J gene	Total	Adm*	DCI*
CASSLIGVSSYNEQFF	TCRBV07-09	TCRBJ02-01	175	1.76	0.87
CASSPPGRGNGYTF	TCRBV28-01	TCRBJ01-02	140	1.23	0.97
CASSLGTGSYNEQFF	TCRBV07-08	TCRBJ02-01	53	0.46	0.37
CASSILGHTGELFF	TCRBV28-01	TCRBJ02-02	35	0.35	0.18
CSAIGDNEQFF	TCRBV20-01	TCRBJ02-01	34	0.28	0.26

Table. Comparison of the clonotype abundance distribution in each patient between the day of admission (Adm) and delayed cerebral ischemia (DCI).

Read frequency (%)	No. 1		No. 2		No. 3		No. 4		No. 5	
	Adm	DCI	Adm	DCI	Adm	DCI	Adm	DCI	Adm	DCI
≥ 5	1	0	0	0	1	1	0	0	0	0
0.5≤ – < 5	8	8	2	0	8	7	2	0	6	4
0.05≤ – <0.5	391	136	10	9	119	60	54	27	60	43
0.005≤ – <0.05	2064	2641	848	1139	3524	8005	4064	4101	4510	3522
Total	2464	2785	860	1148	3652	8073	4120	4128	4576	3569
